# Reviewer acknowledgement 2012

**DOI:** 10.1186/1472-6823-13-6

**Published:** 2013-03-08

**Authors:** Tim Shipley

**Affiliations:** 1 BioMed Central, 236 Gray's Inn Road, WC1X 8HB, London, United Kingdom

## Abstract

**Contributing reviewers:**

The editors of BMC Endocrine Disorders would like to thank all our reviewers who have contributed to the journal in Volume 12 (2012).

## 

Arnold Lohaus

Germany

Mitra Mahdavi-Mazdeh

Iran

Edoardo Mannucci

Italy

Haggi Mazeh

Israel

Pascal Meier

Switzerland

Kazuki Mochizuki

Japan

Peter Mol

Netherlands

Leila B. Moreira

Brazil

Ahmed Mostafa

Egypt

Samiul Mostafa

United Kingdom

Tomoaki Murakami

Japan

Satoshi Murao

Japan

Yoshinori Nagai

Japan

Tomoko Nakagami

Japan

Johnnie Oli

Nigeria

Altan Onat

Turkey

Katharine Owen

United Kingdom

Orhan Veli Ozkan

Turkey

Gopi Krishna Panicker

India

Francisca Pérez-Llamas

Spain

Patricia Perez-Matute

Spain

Lawrence Perlmuter

United States of America

Mark Peyrot

United States of America

Michael Piagnerelli

Belgium

Lars Pieper

Germany

Frans Pouwer

Netherlands

Bhargav Prk

India

Csilla Putz-Bankuti

Austria

Chris Rayner

Australia

Dominic Reeds

United States of America

Simon Rees

United Kingdom

Marc Rendell

United States of America

Anja Rinke

Germany

Kjersti S. Ronningen

Norway

Paolo Rossetti

Spain

Gian Paolo Rossi

Italy

Marek Ruchala

Poland

Guy Rutten

Netherlands

George Sakorafas

Greece

Renata Saucedo

Mexico

Roland Schweizer

Germany

Teresa Maria Seccia

Italy

Alissa Segal

United States of America

Veerasamy Seshiah

India

Emanuela Setola

Italy

James Shaw

United Kingdom

Gunnar Sigurdsson

Iceland

Guenther Silbernagel

Germany

Stefan Söderberg

Sweden

Kate Steinbeck

Australia

Jeroen Struijs

Netherlands

Abd Tahrani

United Kingdom

Yusuf Tamam

Turkey

Bee Tan

United Kingdom

Fatih Tanriverdi

Turkey

Birger Thorsteinsson

Denmark

Christos Toumpanakis

United Kingdom

Armando Tovar

Mexico

Eftihios Trakakis

Greece

Apostolos Tsapas

Greece

Serap Turan

Turkey

Stefanos Tyrovolas

Greece

Yoshihisa Urita

Japan

Luca Valenti

Italy

Valentina Vicennati

Italy

Raquel Villegas

United States of America

Eva Vivian

United States of America

Yvonne Winhofer

Austria

Daisuke Yabe

Japan

Tao Yang

China

John Yim

United States of America

Kazeem Yusuff

South Africa

Nicola Zammitt

United Kingdom

Jin-An Zhang

China

Anca Zimmermann

Germany

## Pre-publication history

The pre-publication history for this paper can be accessed here:

http://www.biomedcentral.com/1472-6823/13/6/prepub

